# Correlation between CD44 and membrane fluidity—a study on biopsies of high-grade serous ovarian tumor: Correlation between CD44 and membrane fluidity

**DOI:** 10.3724/abbs.2022190

**Published:** 2022-12-22

**Authors:** Jun Shi, Yang Yang, Wei He, Munika Moses, Yi-hua Gu, Ningli Li, Wen Di

**Affiliations:** 1 Shanghai Institute of Immunology Shanghai Jiao Tong University School of Medicine Shanghai 200025 China; 2 Department of Obstetrics and Gynecology Ren Ji Hospital School of Medicine Shanghai Jiao Tong University Shanghai 200127 China; 3 Shanghai Institute for Biomedical and Pharmaceutical Technologies Shanghai 200032 China; 4 Department of Pathology Ren Ji Hospital School of Medicine Shanghai Jiao Tong University Shanghai 200127 China; 5 Institut Pasteur of Shanghai Chinese Academy of Sciences Shanghai 200031 China

The functioning of the cell plasma membrane depends on its lipid and protein compositions [1]. Changes in membrane physiochemical characteristics, such as fluidity, are associated with normal cellular physiology [2] and tumor malignancy [3]. As an extensively distributed cell surface marker, CD44 is implicated in the pathogenesis and progression of many cancerous cells, including ovarian cancer cells [4]. A previous study on a cell model indicated that the level of CD44 might be correlated with the fluidity of the plasma membrane [5]. However, no study has been reported on either the status of plasma membrane fluidity or the levels of CD44 in human tumors. In the present report, we studied the status of membrane fluidity and the level of CD44 in normal ovary epithelium and stage III high-grade serous ovarian cancer (HGSOC), a common epithelial ovarian cancer [6]. The main methods are described in Supplementary Data.

By using confocal Raman spectroscopy, we first determined membrane fluidity in the tissue biopsies ( Supplementary Table S1). The spectra of normal ovary epithelium (NOE) and HGSOC were acquired in the Raman shift region from 500 to 3500 cm ^–1^ ( Figure 1A) using the same experimental parameters. Sixteen spectra corresponding to NOE and 36 spectra of HGSOC were acquired from five or six different regions of each biopsy ( Supplementary Figure S1). Raman spectra recorded vibrations of the nucleic acids, proteins, and lipids. Molecular assignments for major peaks are listed in Supplementary Table S2, according to a previous report [7].

**Figure 1:**
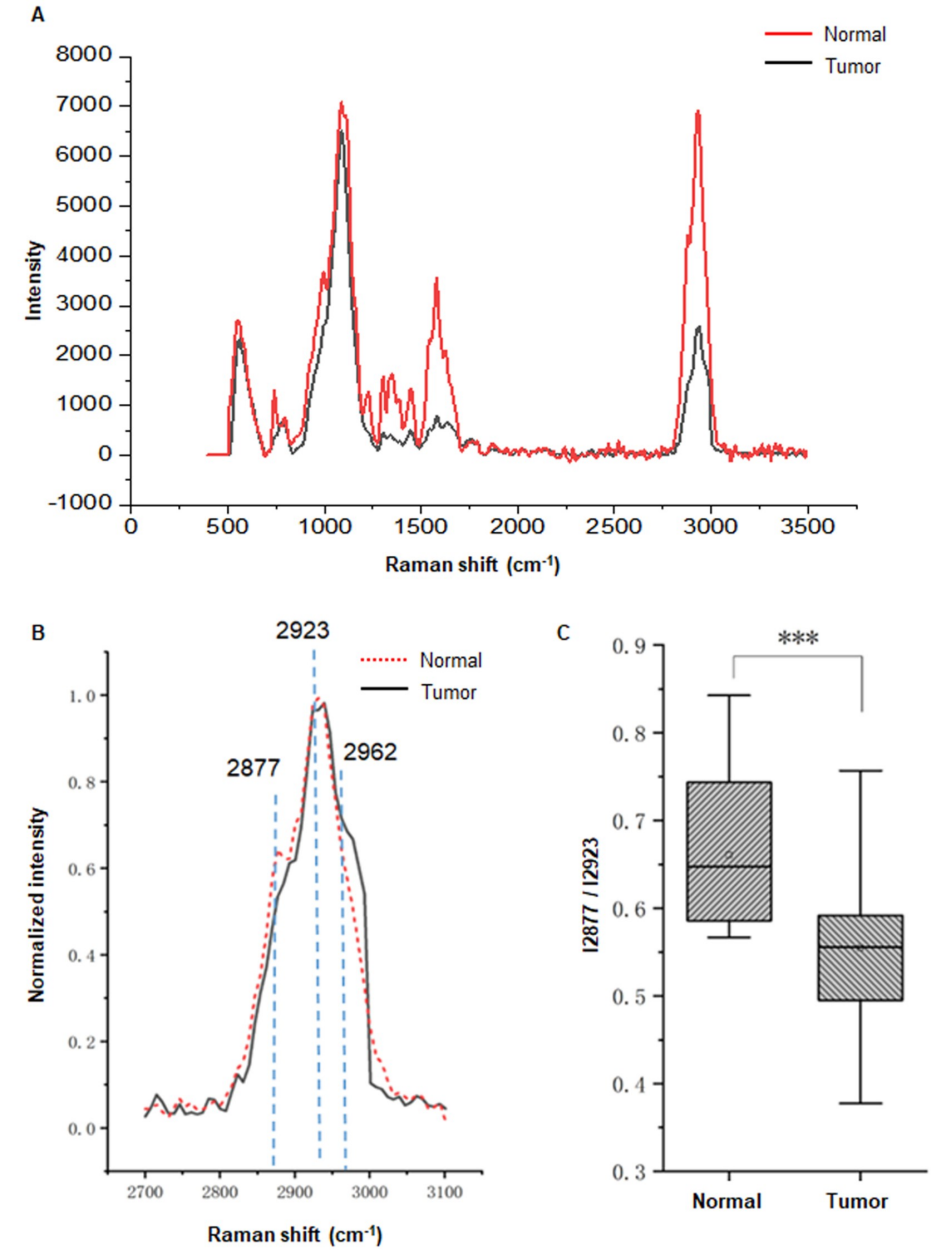
Figure 1 Mean Raman spectra of ovary specimens (A) Mean spectrum of tumors (6 specimens, 31 spectra) or normal ovary epithelium (3 specimens, 16 spectra) from Raman shift of 500 to 3500 cm –1. For a better presentation, the two mean spectra are vertically shifted. (B) Spectrum of normal ovary epithelial (red dotted) or ovary tumor (black solid) was presented in the region from Raman shift of 2700 to 3100 cm –1. Spectra are normalized by the intensity of the maximum peak value in each spectrum. Numbers above peaks correspond to the peak positions in a Raman shift of cm –1. (C) The ratio I2877/I2923 reflects the ordering of the lipid phase in the specimens, and higher ratio means lower membrane fluidity. *** P<0.001.

To study the membrane fluidity of the ovary specimens, we analyzed Raman spectra in the high-frequency region (2700–3100 cm ^–1^) of biopsies. The spectra of ovarian specimens were composed of main peaks at 2877, 2923, and 2962 cm ^–1^ ( Figure 1B), corresponding to =CH2 asymmetric, –CH3 symmetric, and –CH3 asymmetric stretching vibrations, respectively, which are known to come from lipids [8]. In this study, the ratio of intensity at peaks 2877 cm ^–1^ and 2923 cm ^–1^ (I2877/I2923) was used to indicate the ordering of the lipid phase. An increase in this ratio indicates a higher ratio of lipids in the trans conformation, which makes the membrane less fluid [9]. When the ratio I2877/I2923 was used to evaluate the fluidity of ovarian cell membranes ( Figure 1C), we observed a decrease in these ratios, indicating an increase in the membrane fluidity in HGSOC compared with NOE, *P*<0.001.

We next investigated the expression of ovarian cancer biomarkers and CD44 ( *n*=6) in HGSOC using multiplex immunohistochemistry ( Supplementary Figure S2A, Supplementary Table S3). Compared with NOE ( *n*=3), the proportion of tumor biomarker PAX8, P53, CA125 and CK7- or CD44-positive cells was significantly higher in HGSOC than in NOE, while the level of tumor biomarker WT1 was higher in HGSOC than in NOE without statistical significance ( Supplementary Figure S2B, Supplementary Table S4). Since CD44 is also expressed in immune cells, we investigated the proportion of cells expressing both the cancer biomarker CA125 and the membrane fluidity-related protein CD44 ( *n*=6) in HGSOC. Compared with NOE ( *n*=3), the proportion of CA125/CD44-positive cells was significantly higher in HGSOC than in NOE ( 21.30%±23.76% *vs* 0.11%±0.05%, *P*<0.05), which indicated a higher expression level of CD44 in HGSOC tumors ( Figure 2).

**Figure 2:**
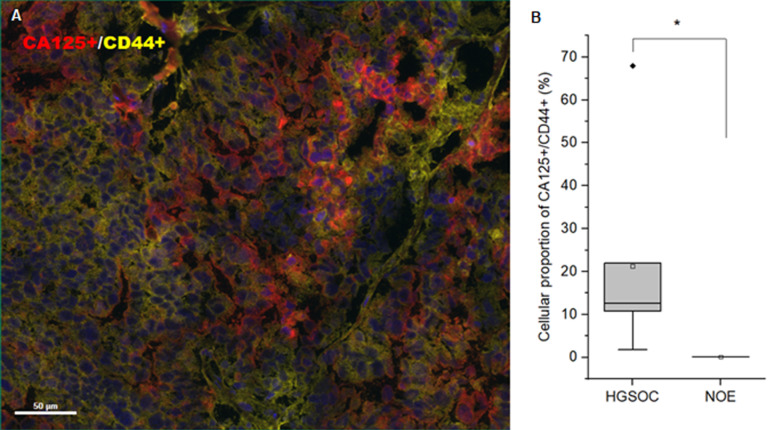
Figure 2 CD125+/CD44+ cells in HGSOC tumors (A) Representative multiplex immunohistochemistry of HGSOC tumors ( n=6) with CD125 and CD44. (B) Comparison of the proportion of CD125+/CD44+ cells between HGSOC ( n=6) and NOE ( n=3). * P<0.05.

In the present study, with *in situ* techniques such as confocal Raman microscopy and mHIC, we determined changes in membrane fluidity and the expression level of CD44 in stage III HGSOC. Compared with NOE, we observed an increase in membrane fluidity correlated with an increase in the CD44 level of HGSOC, which is consistent with shedding of CD44 in the cell model with decreased membrane fluidity induced by increased unsaturated fatty acids [5].

## Supporting information

238Supplementary_Data_-12

## Supplementary Data

Supplementary data is available at *Acta Biochimica et Biophysica Sinica* online.
