# Supplementary material for: Correlation between CD44 and membrane fluidity—a study on biopsies of high-grade serous ovarian tumor: Correlation between CD44 and membrane fluidity
Source: Acta Biochim Biophys Sin (Shanghai). 2022 Dec 22;54(12):1928–30. doi: 10.3724/abbs.2022190 (PMC10157600; doi:10.3724/abbs.2022190)
Supplement: 238Supplementary_Data_-12 [file 238Supplementary_Data_-12.pdf]

Supplementary Data

Materials and Methods

Patients

This study was carried out in compliance with the Helsinki Declaration, with an approved protocol from the Ethics Committee of Ren Ji Hospital, an affiliate of Shanghai Jiao Tong University, School of Medicine (KY2020-047). Patients without previous treatment for high-grade serous ovarian cancer (HGSOC) were recruited at the time of surgery.

Each patient's information is listed in Supplementary Table S1. Nine patients were recruited: six were in stage III of HGSOC, while three with other diseases but normal ovary. Based on results, there was no significant statistical difference in ages between the two groups of tumor and normal ovary, $P>0.05$.

Tissue samples and processes

Ovaries were obtained from surgical resections and frozen in liquid nitrogen. The frozen tissues were minced into smaller pieces with a 3 μm thickness and stored at –80ºC. The slides were then stained with hematoxylin and eosin (H&E staining, Supplementary Figure S1A,C), and analyzed by a trained pathologist, according to the guidelines of International Federation of Gynecology and Obstetrics (FIGO) and World Health Organization (WHO) [1]. The slides adjacent to the H&E stained slides were used for Raman spectroscopy analysis (Supplementary Figure S1B,D) and multiplex immunohistochemistry staining.

Raman spectroscopy analysis

Confocal Raman spectroscopy was applied in this study, with a 532 nm laser excitation. Laser power was set to 9.8 mW, an objective (100×, NA 0.9; Olympus, Tokyo, Japan) was employed to focus the light on the biopsy surface and backscattered light from the target was collected with an optical grating of 600 g/mm by using an Omni-λ 300 monochromator (Zolix Instruments, Beijing, China) to achieve the spectral range from 500 to 3500 cm^{-1}. The light signals were recorded with an integration time of 20–30 s by an Andor iVac-316 CCD camera precooled to –60ºC (Oxford Instruments, Oxford, UK) using FlexScan software ver. 2.2 (Zolix Instruments). Processes of spectra, such as spike removing and smoothing, were made by using FlexScan software 2.2, while baseline subtraction of spectra, statistical analysis of curve, and calculation of Raman peak intensities were performed using OriginPro 2020 software (OriginLab, Northampton, USA).

Multiplex immunohistochemistry (mIHC) staining

The detailed information on mIHC antigens and their antibodies is outlined in

Supplementary Table S2. Ready-to-use working solutions with the first antibody against biomarker of ovary cancer CK7, CA125, P53, PAX8, or WT1 [2] were purchased from Maixin Biotechnology (Fujian, China), and the antibody against CD44, a membrane fluidity related protein [3], was purchased from Cell Signaling Technology (Shanghai, China).

The tumor and normal tissues were assessed with Akoya Opal^{TM} seven-color fluorescent platform. Frozen minced slides were stained with Opal Polaris 7 Color Automation IHC Detection Kit (Akoya Biosciences, Marlborough, USA) for simultaneous detection of the cell nucleus (DAPI, a blue fluorescence stain), and antigen WT1 (Opal 480 channel, a cyan fluorescence), PAX8 (Opal 520 channel, a green fluorescence), CD44 (Opal 570 channel, a yellow fluorescence), P53 (Opal 620 channel, an orange fluorescence), CA125 (Opal 690 channel, a red fluorescence) and CK7 (Opal 780 channel, a purple fluorescence).

The fluorescent signals on slides were recorded with the Vectra Polaris automated quantitative pathology imaging system (Akoya Biosciences). The images were sequentially spectrally unmixed by Akoya Phenoptics inForm software，ver. 2.6.0 (Akoya Biosciences). The cellular quantification was also performed by inForm software. The fluorescent signal of DAPI was used to define each cell. The normalized fluorescent intensity in each channel was then used to set up a positive threshold for each antigen. The cellular proportion was calculated as the ratio of the number of antigen-positive cells to the total cell number.

Statistical analysis

Data are presented either as the mean ± SEM. Statistical data were analyzed using OriginPro 2020 (OriginLab). The student’s *t*-test was used for group comparisons, P<0.05 was considered statistically significant.

References

1. WHO. Female Genital Tumours. 5th ed. International Agency for Research on Cancer; Lyon, France: 2020. Classification of Tumours Editorial Board.
2. Köbel M, Kang EY. The Evolution of Ovarian Carcinoma Subclassification. *Cancers* 2022, 14, 416.
3. Joensuu H , Klemi PJ , Toikkanen S , *et al*. Glycoprotein CD44 expression and its association with survival in breast cancer. *American Journal of Pathology*, 1993, 143(3):867-874.

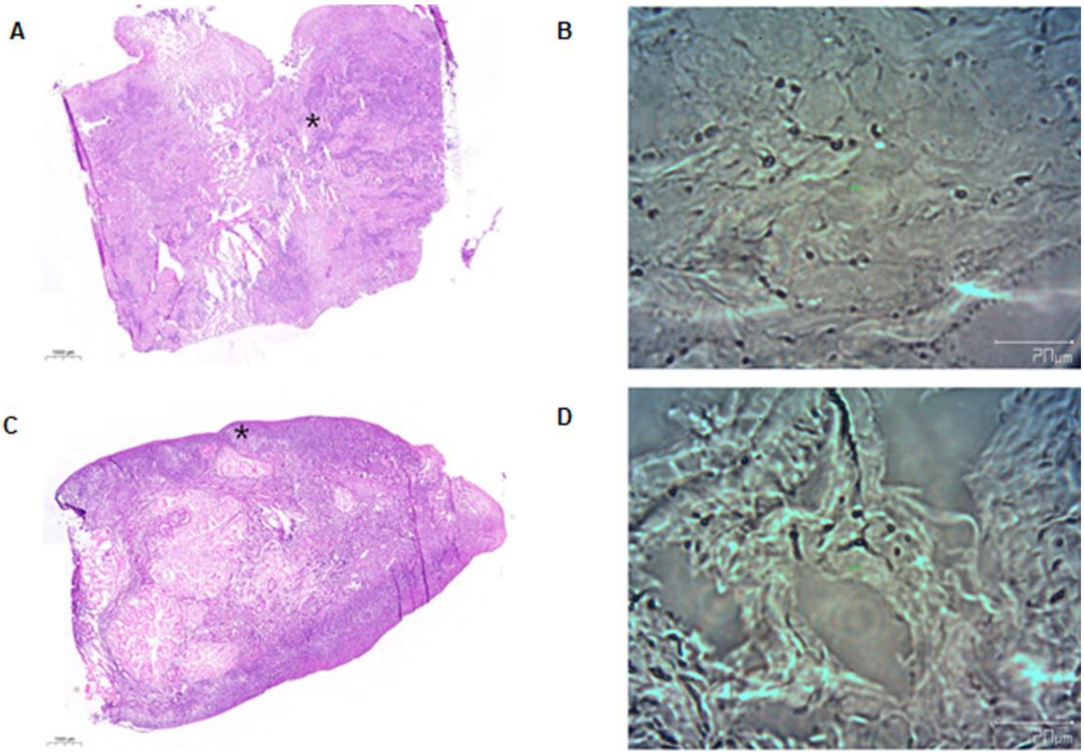

Supplementary Figure S1. Representative hematoxylin and eosin (HE) staining of frozen slices of HGSOC or normal ovarian tissues (A,C) HE staining of HGSOC tumors, and normal ovarian tissues (CaseViewer ver. 2.4, 3DHISTECH Ltd., Hungary), respectively; (B,D) Views of HGSOC tumors, and normal ovarian tissues under confocal Raman microscopy (Zolix Instruments), respectively. Asterisk marks the position where the view is shown under confocal Raman microscopy. Cross marks the position where the Raman spectrum is acquired. Scale bars: 1000 μm (A,C), 20 μm (B,D).

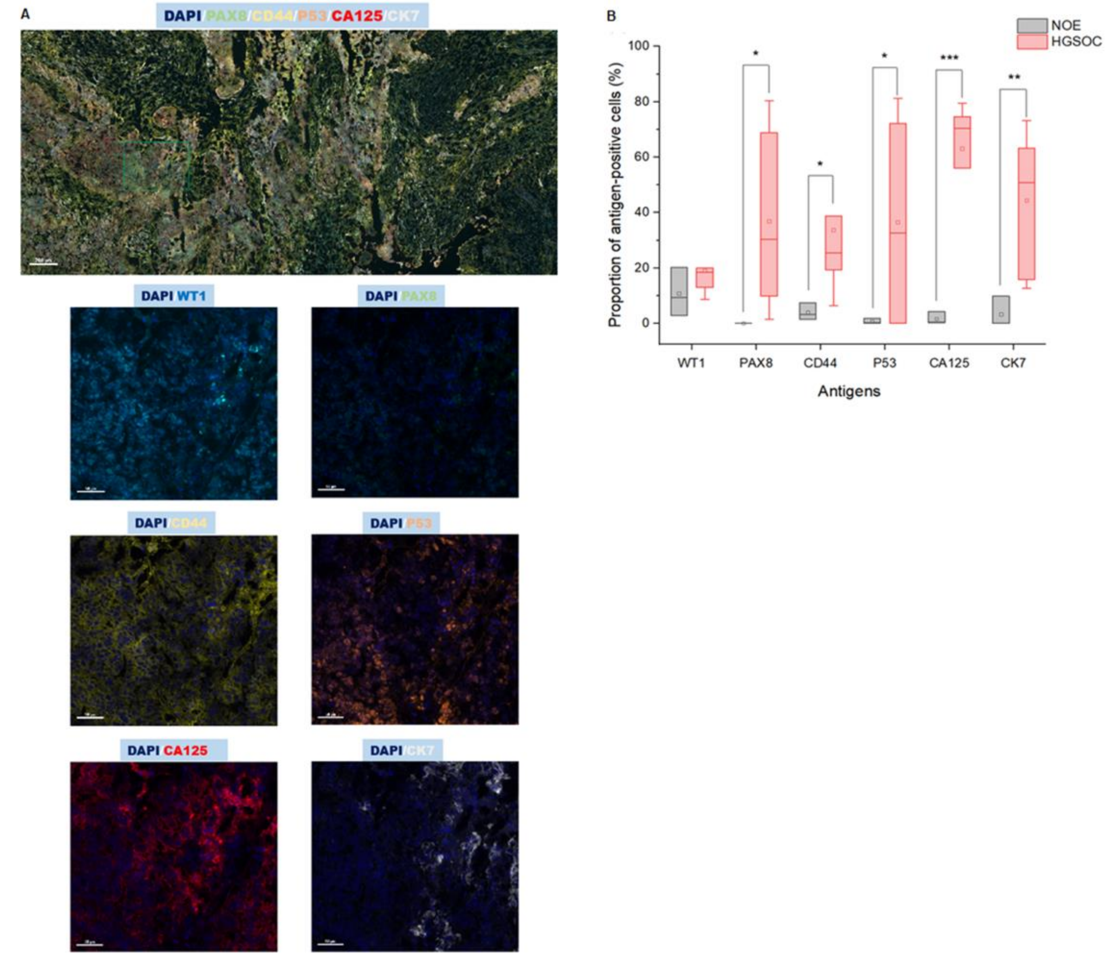

Supplementary Figure S2. Representative multiplex immunohistochemistry of HGSOC tumors (A) The representative multiplex immunohistochemistry of HGSOC tumors (*n*=6) with ovarian biomarkers and CD44, a membrane fluidity-related protein. (B) Comparison of proportion of single antigen positive cells between HGSOC (*n*=6) and NOE (*n*=3). $*P<0.05$, $**P<0.01$, $***P < 0.001$. Scale bars: 200 μm (top), 50 μm (the rest).

Supplementary Tables S1. Patient characteristics

NO	Age	Stage	Degree of differentiation	Pathologic diagnosis
1	65	III	III	Adenocarcinoma III
2	46	III	III	Adenocarcinoma III
3	54	III	III	Adenocarcinoma III
4	55	III	III	Adenocarcinoma III
5	80	III	III	Adenocarcinoma III
6	64	III	III	Adenocarcinoma III
7	59			Normal
8	61			Normal
9	13			Normal

There was no statistically significant difference in ages between the two groups, ovary cancer and normal ovary, $P>0.05$.

Supplementary Table S2. Raman spectrum peak assignment

Raman Shift of Peak (cm^{-1})	Assignment	Main Contribution from Molecules
718	Choline	Lipid
740	C-S trans, tryptophan	Protein
786	U,C,T ring breathing	Nucleic acid
932	Glutathione,C-C backbone stretch	Lipid or protein
995	Phenylalanine,C-C skeletal	Protein
1086	C-N stretch	protein
1127	C-N stretching; C-C stretching	Protein or lipid
1158	C-C, C-N stretch	Protein
1302	C-H deformation	Lipid
1553	Tryptophan	Protein
1579	Pyrimidine ring	Nucleic acid
2877	v_{as}（=CH2）	Lipid
2923	v_s （-CH3）	Lipid
2962	v_{as}（-CH3）	Lipid

Supplementary Table S3. Multiplex immunohistochemistry antigens

Antigen	Antibody		Dilution	Incubation time	TSA Dyes
	Catalog	Clone			
Nucleus	DAPI			1 h	DAPI
WT-1	MAB-0678	MX012	1:1	1 h	Opal 480
Pax-8	RMA-0817	EP298	1:3	1 h	Opal 520
CD44	CST #3570T		1:100	2 h	Opal 570
P53	MAB-0674	MX008	1:1	1 h	Opal 620
CA125	MAB-0777	M11	1:2	1 h	Opal 690
CK7	Kit-0021	OV-TL12/30	1:1	1 h	Opal 780

Supplementary Table S4. Proportion of antigen-positive cells (%)

	Antigens					
	WT1	PAX8	CD44	P53	CA125	CK7
Ovary cancer	19.02±9.18	36.90±32.07	33.72±28.34	36.52±40.07	63.17±18.91	44.45±24.88
NOE	10.87±8.71	0.07±0.07	4.04±3.14	0.73±0.99	1.66±2.21	3.35±5.70
P (Student's *t* test)	0.132	0.019	0.026	0.040	0.000	0.004
